# Motor and Perceptual Recovery in Adult Patients with Mild Intellectual Disability

**DOI:** 10.1155/2018/3273246

**Published:** 2018-04-23

**Authors:** Mariagiovanna Cantone, Maria A. Catalano, Giuseppe Lanza, Gaetano La Delfa, Raffaele Ferri, Manuela Pennisi, Rita Bella, Giovanni Pennisi, Alessia Bramanti

**Affiliations:** ^1^IRCCS Centro Neurolesi Bonino Pulejo, Via Provinciale Palermo, Contrada Casazza, 98124 Messina, Italy; ^2^Associazione Assistenziale Villa Sandra, Via per Aci Bonaccorsi 16, San Giovanni La Punta, 95037 Catania, Italy; ^3^Oasi Research Institute-IRCCS, Via Conte Ruggero 73, Troina, 94018 Enna, Italy; ^4^Spinal Unit, Emergency Hospital “Cannizzaro”, Via Messina 829, 95126 Catania, Italy; ^5^Department of Medical and Surgical Sciences and Advanced Technologies, Section of Neurosciences, University of Catania, Via S. Sofia 78, 95123 Catania, Italy; ^6^Department of Surgery and Medical-Surgical Specialties, University of Catania, Via S. Sofia 78, 95123 Catania, Italy

## Abstract

**Introduction:**

The relationship between intellectual disability (ID) and hand motor coordination and speed-accuracy, as well as the effect of aging on fine motor performance in patients with ID, has been previously investigated. However, only a few data are available on the impact of the nonpharmacological interventions in adult patients with long-term hand motor deficit.

**Methods:**

Fifty adults with mild ID were enrolled. A group of thirty patients underwent a two-month intensive ergotherapic treatment that included hand motor rehabilitation and visual-perceptual treatment (group A); twenty patients performing conventional motor rehabilitation alone (group B) served as a control group. Data on attention, perceptual abilities, hand dexterity, and functional independence were collected by a blind operator, both at entry and at the end of the study.

**Results:**

After the interventions, group A showed significantly better performance than group B in all measures related to hand movement from both sides and to independence in activities of daily living.

**Discussion:**

Multimodal integrated interventions targeting visual-perceptual abilities and motor skills are an effective neurorehabilitative approach in adult patients with mild ID. Motor learning and memory-mediated mechanisms of neural plasticity might underlie the observed recovery, suggesting the presence of plastic adaptive changes even in the adult brain with ID.

## 1. Introduction

Intellectual disability (ID) is the most common development disorder, affecting approximately 1% of the general population in Europe [1]. ID typically impairs psychomotor skills and limits the abilities of daily living. A number of factors are associated with ID, including genetic and congenital causes (such as Down's syndrome (DS)), toxin exposure, infections, prematurity, birth injuries, and perinatal hypoxia, although most cases are of unknown etiology. Life expectancy has recently risen, but it still remains lower than that of the general population [2]. Moreover, epilepsy, behavioral disorders, and other medical diseases are frequent comorbidities and cause need for polypharmacotherapy and long-term social and health care [3]. Finally, adults with ID (namely, those with DS [4]) show a higher risk to develop dementia [5], which is characterized by a frequent and early tendency to lose independence and be institutionalized.

Subjects with ID are commonly described as being “clumsy” and with poor motor coordination, difficulty in both fine and gross movements and motor planning. The combination of cognitive and long-standing sensory-motor deficits generally causes a variable degree of upper limb disability, which impairs even common activities of daily living, such as grasping small objects or the hand-finger movements. In addition, these patients tend to have a greater prevalence of physical decline compared to the aged general population, especially in terms of motor speed and accuracy of purposeful movements [6]. Recently, the relationship between ID and motor impairment concerning the areas of coordination, hand dexterity, and movement speed has attracted increasing attention [7–10]. In particular, the analysis of measures of reaction time and finger dexterity indicates that people with DS have more difficulty in performing fine movements [11]. On the other hand, it is known that normal aging interferes with fine motor performances [12, 13], and, therefore, the age-related decline of motor performance might be more pronounced in people with ID for tasks that are under perceptual or motor constraints, such as movement accuracy, speed, and reaction time. This is in line with the evidence that ID subjects usually display limitations in functional use of the hands, ranging from a mild deficit of in-hand manipulation to a severe impairment that makes grasping or holding an object even impossible [14].

As known, movement control and motor learning are driven by multiple sensory inputs. For instance, when the arm control is impaired, vision and other sensorial modalities, such as proprioception, can all support the arm movements and guide the necessary adjustments for correcting the errors. In this context, the spatial perception has a pivotal role in the development of motor skills and in particular in the Euclidean representation of the environment. This refers to a subtype of intuitive or natural geometry, which is largely a cross-cultural universal ability resulting from inherent properties of the human mind [15, 16]. More in detail, motor achievements may be integrated in the domains of tactile perception and depth perception. Usually, there is a high degree of concordance between the developmental stage in which certain perceptual sensitivities unfold and the corresponding onset of motor abilities [17, 18]. In patients with ID, both motor and perceptual developments are known to be impaired [19]. More recently, an altered perception of Euclidean geometry has been described in a group of children with symptoms of nonverbal disability, highlighting the relevance of the Euclidean perception also in cognitive tasks [20]. In this view, the rehabilitative-induced enhancement of the spatial perception might improve the efficacy of hand motor coordination during the object manipulation [21]. Similar approaches were previously and successfully applied also in patients recovering from mild-to-severe brain injury, as well as in a large cohort of children with mild ID [21, 22].

Based on this theoretical background, the aim of the present study was to assess and compare clinical data of motor dexterity in a group of adult patients with mild ID before and after ergotherapic activities involving Euclidean perception. This is to evaluate the efficacy on fine movement recovery and to indirectly probe any plastic change occurring in the adult brain with ID.

## 2. Materials and Methods

### 2.1. Participants

A group of 50 adult patients attending the Rehabilitation Department of the “Associazione Assistenziale Villa Sandra” in San Giovanni La Punta (Italy) were enrolled. All subjects met the diagnostic criteria of ID according to the American Association on Intellectual and Developmental Disabilities [23] and the Diagnostic and Statistical Manual of Mental Disorders-IV Edition (DSM-IV) [24]. They also showed significant impairment of global mental abilities, significant deficit of one or more areas of adaptive behaviour across multiple environments, and evidence that these limitations became apparent in their childhood or adolescence. Patients with mild ID (IQ = 50–69), rated by the intelligence quotient (IQ) scores defined by the Wechsler Abbreviated Scale of Intelligence [25] were included. Moreover, after a careful clinical evaluation, patients with IQ 70–79 (the so-called “borderline status”) were also included due to their severe impairment in adaptive functioning.

Patients were divided into two groups: group A (30 patients, 15 females; median age 36.8 years, range 22–53 years; median IQ = 56.5, range 50–76), undergoing an intensive ergotherapic treatment that included both motor hand rehabilitation and cognitive-perceptual treatment, and group B (20 patients, 10 females; median age 38.7 years, range 27–45; median IQ = 55.0, range 50–70), performing conventional motor rehabilitation alone. All patients continued to receive their medical treatment, as well as usual health and recreational activities. Demographic and clinical characteristics of both groups at baseline are summarized in Table 1.

The condition underlying ID was unknown or not reported from one-third to one-half of the cases, whereas the remaining subjects were affected by DS. Patients with a severe ID, those who were unable to understand simple verbal orders, and subjects with a history of major psychiatric disorders or other neurological diseases (including dementia), those with acute or chronic not compensated medical illnesses, endocrinopathies, alcohol or drug abuse, and auditory or visual deficits, were excluded.

The study was approved by the local Ethics Committee and performed in accordance with the ethical standards of the Declaration of Helsinki in 1964 and its later amendments. Patients were enrolled after signing the informed consent.

### 2.2. Clinical Assessment

Clinical features were collected both at the entry of the study and after a period of two months of the interventions. The evaluation of patients with ID was a complex multifaceted process performed by both trained therapist and skilled physician. It encompassed an initial interview, followed by an informal assessment/clinical observation lasting from three to four hours, a neurological exam, and a formal assessment of cognitive, perceptual, and motor abilities using standardized scales, as described below. For some clinical variables, such as attention or praxis, a qualitative score was assigned from 0 to 3 on the basis of pure clinical observation (0 = normal; 1 = mild impairment; 2 = moderate impairment; and 3 = severe impairment). Similarly, the Euclidean perception of the space was scored from 0 to 2 (0 = normal perception; 1 = partial perception; and 2 = no perception).

Gross motor function was classified using the Italian version of the Gross Motor Function Classification System (GMFCS), expanded and revised [26]. GMFCS was originally developed for evaluating the severity of gross motor dysfunction of spontaneous movements, trunk control, and walking ability in children with cerebral palsy and other ID-associated disorders [27]. GMFCS is a 5-level classification system with an increasing gradient of gravity that differentiates patients with cerebral palsy based on their age current gross motor abilities and need for assistive technology and wheeled mobility. Patients classified in the level I can generally walk without restrictions, although tend to be limited in some of the more advanced motor skills; those classified at the level V are generally very limited in their ability to move themselves around, even with the use of assistive technology. This grading system has shown to be reliable across observers and with increasing age [28].

Bimanual Fine Motor Function (BFMF) is a classification of the hand function in children with cerebral palsy based on a five-level scale, whereby level I describes the best and level V the most limited function [29, 30]. BFMF can usefully describe and classify the fine motor capacity, providing additional information when used together with the Manual Ability Classification System (MACS) [31]. The latter is a classification of how patients with cerebral palsy use their hands when handling objects in daily activities with a focus on the use of both hands together, and it is extensively used in both clinical practice and research setting, providing relevant and reliable information on manual performance [31, 32]. As mentioned above, MACS also includes 5 levels of severity, the level I being the least affected (difficulty only in tasks needing speed and accuracy), and the level V the most impaired (not able to handle objects and severely limited abilities even for simple actions).

Data on hand motor dexterity were collected by using the Nine Hole Peg Test (NHPT), which is a widely validated measure used in several disorders [33, 34]. NHPT requires participant to repeatedly place and then remove nine pegs into nine holes, one each time, as quickly as possible. Score is influenced by muscle strength, tactile sensitivity of the thumb, and presence of intention tremor. The time needed to complete the task in seconds is the most frequently reported metric in the literature. In addition to the motor functioning, NHPT probes also the hand-eye coordination in patients with ID [35].

All evaluations were performed in a dedicated and quiet room, with a standardized set of verbal instructions followed by a demonstration of the task. Hand dominance was determined by the Edinburgh Handedness Inventory [36], and the dominant hand was the first to be tested. Functional status was assessed by the Activity Daily Living (ADL) and the Instrumental Activity Daily Living (IADL) scales.

### 2.3. Ergotherapic Activities

**Figure 1:**
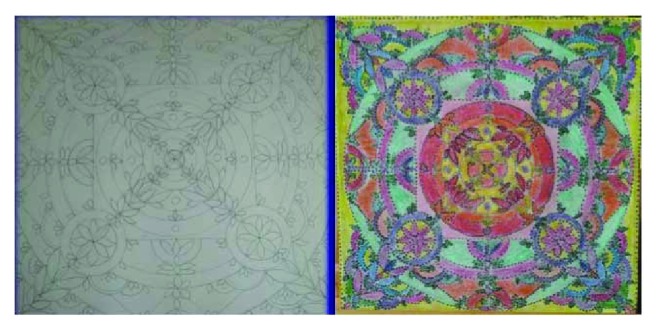
Example of a mandala figure (left side: template; right side: completed figure).

Group A underwent occupational therapy targeting the rehabilitation of Euclidean spatial perception and hand motor functions through a graphic-motor protocol using a geometric pattern resembling the mandala figures (Figure 1) [17]. The basic form of most of the graphics was a square with four gates containing a circle with a center point. The use of this protocol was based both on the ecological approach of perceptual learning as a process of seeing the differences in the perceptual field around an individual and on the Piaget's theory of perceptual development [17, 18]. Every picture used in this study was designed considering the following parameters: ability to recognize an open space from a closed one; curved visual capability; simple or complex structured visual skills in relation to the figure; Euclidean perception; and ability to monitor the visual representation of the graphic segment. Specifically, during the activities, the following skills were established: ability to recognize a center point and the main parts of the picture; capacity to discriminate different configurations; ocular-manual coordination; ability to adequately place topological parameters; and ability to differentiate chromatic tracks.

Each patient, supervised by a skilled operator, started by composing the figure from simple sequences of lines intersecting in the canvas by obtaining a center. From this focal point, the participant builds simple and flat geometric shapes without the Euclidean representation (first level of ergotherapic protocol). Then, by using colours, the size, distance, shape, and orientation of the surfaces were highlighted (second level). By using a further geometric stratification, the depth of the picture was obtained (third level). In the fourth and last level of the ergotherapic protocol, subjects decorated every single part of the picture on a chromatic basis, providing circular or sinusoid lines. The graphical tools, such as pencils, paint brushes, and watercolours, were chosen based on the graphical ability and the residual capacity to control the imprinted force in the hand and its maintenance during each chronological step.

Conventional motor rehabilitation protocol included a daily session, ranging from 45 to 60 minutes, of progressive resistance/strength-based exercises of the upper limbs. The interventions were delivered by a trained physiotherapist.

### 2.4. Statistical Analysis

Because of the nonnormal distribution of data (assessed by means of the Shapiro-Wilk *W* test), the nonparametric Mann–Whitney test for independent data sets was used to compare data from the two groups. Differences in symptom frequency between baseline and after treatment were evaluated by means of the chi-square test or the Fisher exact test (when any expected frequency was below 5). A *p* level of 0.05 was considered statistically significant.

## 3. Results

At the entry, all patients exhibited impairment of attention, sensory-motor functioning, and Euclidean perception; conversely, they were able to perform gross motor skills (such as running), although balance and coordination were partially limited. Therefore, they were able to walk at home and in outdoor spaces and to climb stairs without the use of railings. As shown in Table 2, patients exhibited a level I according to GMFCS. When considering the fine motor function (i.e., the capacity to grasp, hold, and manipulate objects for each hand separately), they were classified within the level 1 according to BFMF and within the level 2 for group A and 1 for group B according to MACS.

All participants successfully concluded the rehabilitation protocol without the need of any special accommodation. Attention and Euclidean perception, together with different abilities of the hand (such as strength, grasping, palmar movements, handling, and finger holding), significantly improved in group A only (*p* < 0.001). A significant improvement of almost all formal measures of hand movements was observed in both groups, although with better results in patients under the experimental condition. In particular, we observed a better response for measures of bimanual dexterity in group A compared to group B (ΔBFMF: −1.0 versus 0.1, *p* = 0.019; ΔMACS: −1.9 versus −1.3, *p* = 0.018). Finally, a significant amelioration of both IADL and ADL was obtained in group A only (ΔIADL: −1.2 versus 0.4, *p* = 0.037; ΔADL: −1.0 versus 1.0, *p* = 0.008). Data on motor and sensory abilities at the end of the ergotherapic training are shown in Tables 3 and 4.

## 4. Discussion

The main finding of this study shows a significant improvement of attention and Euclidean perception together with different motor abilities of the hand in adult patients with mild ID undergoing a two-month training of ergotherapic treatment focusing on visual-perceptual and hand motor functions. In the experimental group, we also observed a better response for measures of bimanual dexterity and independence in activities of daily living. As previously reported [37], adults with ID score poorly in manual tasks that recruit motor and visual abilities, being this considered as a consequence of the ID per se and an acquired motor or visual impairment secondary to the brain aging. In this context, the observed results underline that a multimodal integrated rehabilitative approach based on both physical and visual-perceptual training in a dedicated center might be more effective than the conventional therapy alone.

In the present study, the recovery of some motor performances meant as a reappropriation of each single component of a complex movement and perceptual ability. Indeed, to complete the Euclidean task, patients were required to optimize their gross and fine motor praxis, to have a proper exploration of the space, and to correctly quantify the force to be impressed on their hand. In addition, this type of intervention significantly motivated participants and tended to keep constant their attentive focus, suggesting that the “hand-eye-mind” pathway may act as a compensatory mechanism for the ID-associated deficits. Notably, all these steps are preparatory and necessary to possibly gain further recovery of increasingly complex abilities. Hand-eye coordination is a complex cognitive ability as it calls for a strong relationship between visual and manual motor systems. This integrated relationship finely coordinates motor responses of both eye and hand to produce controlled, rapid, and accurate movements. This system is of crucial importance for the normal child development, but it is also relevant for activities of daily living in adult people. Indeed, deficit of ocular or manual control has been studied also after an acquired brain injury [38].

From a neuroanatomical perspective, motor learning requires the development and retention of several skills, depending on the structural and functional integrity of the neostriatum and the cerebellum. These areas are also supported by a large cerebral network modulating both ocular and manual motor control. Indeed, the anatomophysiology of the human eye movement control is due to a wide interconnected system of cortical and subcortical structures that includes the frontal and parietal eye fields, the prefrontal cortex, the supplementary eye field, the basal ganglia, and the cingulate eye field [39]. Motor and premotor cortices, together with the somatosensory cortex, the cerebellum, and the basal ganglia, are all engaged in reaching an optimal motor control [40–42].

Interestingly, the study provides evidence that specific processes of motor learning and memory-mediated plastic mechanisms of recovery might occur also in the adult brain with ID, supporting the role of rehabilitation even for adult people with chronic pathologies. Nevertheless, the approach and setting for this type of patients are rather challenging, often requiring comprehensive services by different rehabilitation professionals to ensure that multidimensional issues can be successfully addressed. Accordingly, patients with ID should be guided by a complex ergotherapic process, through which they can reacquire, totally or partially, the spectrum of cognitive, perceptual, and motor skills that are impaired, from the basic skills to the more complex ones, in the same sequence they were first acquired during the normal development. The goal is to restore the disrupted brain processes underlying motor cognitive operations, in order to promote an accurate and efficient functioning which is based on proper sensory integration and powerful information processing. Finally, an active participation in meaningful and purposeful ergotherapic activities promotes motivation and improves subject's feelings, attitudes, and behaviors.

As known, plastic cortical changes are considered to be the substrate of learning and memory, both in development and aging and in physiological and pathological conditions. Several mechanisms are involved in the induction and modulation of neural plasticity, including phenomena of long-term potentiation and long-term depression, second messenger pathway activation, gene transcription, and morphological changes in neuronal membranes, axons, and postsynaptic cells [42]. Previous studies showed that the impairment of learning and memory in ID might result from a deficient synaptic plasticity due to several pathological processes, such as aberrant protein expression, altered molecular rearrangement, and excitatory-inhibitory neurotransmitter imbalance, eventually leading to maladaptive changes in neuronal circuitry [43–46]. Recently, noninvasive brain stimulation techniques have been used to assess the *in vivo* functional integrity of intracortical neurons and cortical-spinal fibers [47, 48], to probe and monitor the excitability and connectivity of the human brain [49–53], and to modulate neural plasticity or even revert maladaptive plasticity [54–58], thus providing intriguing insights into the pathophysiology and neurochemistry of several neurological and psychiatric disorders [59–65]. These techniques have been successfully applied also in patients with DS, fragile X syndrome, and low-functioning autism [66–68], as well as to promote motor recovery in patients with chronic stroke [69–71]. Overall, these findings open new exciting windows into the noninvasive rehabilitative interventions targeting cortical plasticity and neural connectivity. Finally, relatively little is known on the aberrant plasticity and/or metaplasticity in adults with ID [72, 73]. In this frame, further neurophysiological studies are encouraged to design experimental protocols based on physical activity, cognitive training, and innovative drugs.

The main limitations of this study are the relatively small number of participants and the lack of a follow-up study to prove the long-term effects of the intervention. In addition, although group A was very homogeneous in terms of clinical demographic features and age-matched with controls, it had more patients with moderate ID compared to the other groups; therefore, we cannot exclude that this might have partially influenced the results.

## 5. Conclusions

A combined intervention targeting motor and visual-perceptual skills is clinically and functionally effective in adults with mild ID, suggesting that neuroplastic adaptive changes may take place even in the adulthood of these patients. The underlying mechanisms will be further defined possibly combining different electrophysiological and neuroimaging techniques (such as high-density electroencephalography, transcranial magnetic stimulation, magnetoencephalography, and functional magnetic resonance imaging). Future studies are needed to clarify the impact of rehabilitative interventions on neural plasticity of motor and nonmotor cortical areas, to identify those subjects who would most likely respond to a specific intervention modality, to set up customized protocols, and to establish proper timing of observation and measures of outcome.

## Figures and Tables

**Table 1:** Demographic and clinical characteristics of patients at baseline.

	Group A	Group B
Number and gender	15 F/15 M	10 F/10 M
Median age, years	36.8 (range 22–53)	38.7 (range 27–45)
Median IQ, score	56.5 (range 50–76)	55.0 (range 50–70)
Handedness (R/L)	22/8	15/5

Group A: experimental group; Group B: control group; IQ: intelligence quotient; M: male; F: female; R: right-handed; L: left-handed.

**(a):** 

Clinical evaluation	Group A		Group B	
	Normal	Mild-moderate-severe impairment	Normal	Mild-moderate-severe impairment
Attention	1	8-21-0	1	7-12-0
Visual system	14	16-0-0	16	4-0-0
Auditory system	26	4-0-0	20	0-0-0
Sensory system	29	1-0-0	20	0-0-0
Motor system	21	9-0-0	12	8-0-0
Euclidean perception	0	16-14-0	0	20-0-0
Strength of the hand	4	13-11-2	16	4-0-0
Grasping	8	17-4-1	15	5-0-0
Linear palmar movement	8	18-4-0	16	4-0-0
Circular palmar movement	4	20-6-0	7	11-2-0
Handling	12	15-3-0	17	3-0-0
Finger holding	14	13-3-0	19	1-0-0

**(b):** 

Formal evaluation	Group A			Group B				
	Median	Lower Q	Upper Q	Median	Lower Q	Upper Q	M-W “*U*”	*p*
NHPT3 left	18.2	15.9	21.3	18.8	17.3	20.1	247.0	NS
NHPT2 left	18.8	16.9	22.6	20.5	18.6	22.0	240.0	NS
NHPT1 left	20.8	17.2	22.7	21.7	18.6	22.8	242.0	NS
NHPT3 right	18.3	14.9	22.0	19.6	16.3	22.9	264.5	NS
NHPT2 right	18.6	15.6	23.7	20.8	16.9	24.6	259.0	NS
NHPT1 right	19.7	15.5	28.0	21.2	17.9	25.1	266.0	NS
IADL	7.0	5.0	8.0	5.0	2.0	8.0	211.0	NS
ADL	5.0	4.0	6.0	4.5	3.0	6.0	247.5	NS
MACS	2.0	2.0	2.0	1.5	1.0	2.0	240.0	NS
BFMF	1.0	1.0	2.0	1.0	1.0	2.0	285.0	NS
GMFCS	1.0	1.0	2.0	1.0	1.0	2.0	267.0	NS

Group A: experimental group; Group B: control group; M: male; F: female; R: right-handed; L: left-handed; NHPT: Nine Hole Peg Test; IADL: instrumental activity of daily living; ADL: activity of daily living; MACS: Manual Ability Classification System; BFMF: Bimanual Fine Motor Function; GMFCS: gross motor function classification system; Q: quartile; M-W: Mann–Whitney test; NS: not significant.

**Table 3:** Scores at the end of the rehabilitation protocol in the group A (scores did not significantly change in the group B performing conventional motor rehabilitation alone).

T	Normal	Mild-moderate-severe impairment	Chi-square	*p*
*Attention*				
T0	1	8-21-0	22.5	**<0.0001**
T1	12	14-4-0		
*Visual system*				
T0	14	16-0-0	0	NS
T1	14	16-0-0		
*Auditory system*				
T0	26	4-0-0	^∗^	NS
T1	26	4-0-0		
*Sensory system*				
T0	29	1-0-0	^∗^	NS
T1	29	1-0-0		
*Motor system*				
T0	21	9-0-0	0	NS
T1	21	9-0-0		
*Strength of the hand*				
T0	4	13-11-2	5.97	**<0.05**
T1	12	11-7-0		
*Grasping*				
T0	8	17-4-1	^∗^	**<0.014**
T1	18	11-1-0		
*Linear palmar movement*				
T0	8	18-4-0	^∗^	**<0.043**
T1	18	10-2-0		
*Circular palmar movement*				
T0	4	20-6-0	^∗^	**<0.0018**
T1	17	10-3-0		
*Handling*				
T0	12	15-3-0	^∗^	**<0.0038**
T1	24	5-1-0		
*Fingers holding*				
T0	14	13-3-0	^∗^	**<0.01**
T1	25	4-1-0		
*Euclidean perception*				
T0	0	16-14-0	15.77	**0.0004**
T1	12	12-6-0		

T: timing; T0: baseline; T1: after experimental treatment; NS: not significant; numbers in bold: statistically significant *p* values; ^∗^: Fisher exact test.

**Table 4:** Changes of motor hand functions and independence scores at the end of the protocol.

	Group A			Group B				
	Median	Lower Q	Upper Q	Median	Lower Q	Upper Q	M-W “*U*”	*p*
NHPT3 left	0.0	0.0	0.0	0.0	0.0	0.0	300.0	NS
NHPT2 left	0.0	0.0	0.0	0.0	0.0	0.0	300.0	NS
NHPT1 left	−1.0	−1.0	0.0	0.0	0.0	0.0	110.0	**0.0002**
NHPT3 right	0.0	0.0	0.0	0.0	0.0	0.0	270.0	NS
NHPT2 right	0.0	0.0	0.0	0.0	0.0	0.0	300.0	NS
NHPT1 right	−1.9	−4.2	0.7	0.0	−1.3	2.0	180.5	**0.018**
IADL	−1.2	−3.3	0.7	0.4	0.0	0.7	194.0	**0.037**
ADL	−1.0	−2.4	0.5	1.0	−0.7	1.5	165.0	**0.008**
MACS	−1.9	−3.8	0.0	0.2	−1.3	1.1	166.5	**0.018**
BFMF	−1.0	−2.5	0.6	0.1	0.0	0.5	167.0	**0.019**
GMFCS	0.0	−2.3	2.0	0.0	−0.8	0.8	260.0	NS

Group A: experimental group; Group B: control group; NHPT: Nine Hole Peg Test; IADL: instrumental activity of daily living; ADL: activity of daily living; MACS: Manual Ability Classification System; BFMF: Bimanual Fine Motor Function; GMFCS: gross motor function classification system; Q: quartile; M-W: Mann–Whitney test; NS: not significant; numbers in bold: statistically significant *p* values.
